# COVID-19-Related Stressors and Psychophysical Health Conditions among Italian University Students: A Post Pandemic Insight

**DOI:** 10.3390/healthcare12070752

**Published:** 2024-03-29

**Authors:** Maria Francesca Cattaneo Della Volta, Federica Vallone, Maria Clelia Zurlo

**Affiliations:** 1 Department of Humanities, University of Naples Federico II, 80133 Napoli, Italy; mariafrancesca.cattaneodellavolta@unina.it (M.F.C.D.V.); federica.vallone@unina.it (F.V.); 2 Dynamic Psychology Laboratory, Department of Political Sciences, University of Naples Federico II, 80138 Napoli, Italy

**Keywords:** COVID-19 pandemic, university students, post-pandemic mental disorders, COVID-19-related stressors, psychophysical health outcomes, repeated cross-sectional study

## Abstract

The COVID-19 medical emergency has ended worldwide, yet the psychological impact of these years of unprecedented changes on students’ lives still needs to be deepened. Methods: This study aims to assess and compare COVID-19-related stressors (relationships and academic life; isolation; and fear of contagion) and psychophysical symptoms reported by 637 university students at three times, i.e., April 2020 (*n* = 197), April 2021 (*n* = 200), and April 2022 (*n* = 240). The impact of COVID-19-related stressors on psychophysical symptoms within each time was also tested. Results: In April 2022, perceived isolation and fear of contagion decreased from the peak registered in April 2021, but stress related to relationships and academic life remained high. An ongoing increase in psychophysical symptoms was found. More than 50% of students reported clinical levels of sleep disorders, depression, psychoticism, and interpersonal sensitivity. In April 2022, students still perceiving stress related to relationships and academic life and isolation were at risk for anxiety, somatization, and sleep disorders. Students still perceiving stress related to fear of contagion were also at risk for depression, obsessive–compulsive symptoms, and psychoticism. Conclusion: The findings emphasized the long-lasting effects of COVID-19-related stress on students’ psychophysical health. Interventions must aim at supporting students in dealing with the complex post-pandemic adjustment process.

## 1. Introduction

In the last decades, research attention was progressively given to university students’ wellbeing, since both the prevalence and the awareness of poor mental health registered among students worldwide have increased over time [1]. In this direction, an international survey developed by the World Health Organization [2] has underlined that more than 30% of university students met the diagnostic criteria for at least one common mental disorder. Specifically, several studies highlighted remarkable levels of stress and post-traumatic stress disorders, anxiety, depression, and obsessive–compulsive disorders [1,3,4,5,6]. 

In the same direction, more recently, a systematic review and meta-analysis estimating the prevalence of students’ mental health problems suggested that university students worldwide frequently reported feelings of isolation, social disconnection, and loneliness, as well as remarkable levels of stress, anxiety, suppressed anger, and sleep disorders [7]. However, the review above mentioned has primarily underlined alarming levels of depression, i.e., the prevalence of depression among university students ranged from 10% to 58%, and these levels are significantly higher than those registered in the general population [5,8,9]. 

However, it should also be noticed that the transition phase from secondary to tertiary education represents, in itself, a high-risk time for students, given the significant developmental changes they have to deal with (e.g., the transition to adulthood; the adjustment to new demands/roles in the family domain, in relationships with peers, as well as in academic life), but also considering that the age of onset of several mental health disorders, including psychoses and schizophrenia, which commonly co-occur with the beginning of college/university life [1,10,11]. 

Within this already complex portrait, over the last several years, university students have been additionally challenged by the spread of the COVID-19 pandemic, which has imposed several drastic modifications to their customary life [12,13], potentially entailing significant levels of stress related to the changes in their relational and academic life (i.e., relationships with family members and peers, as well as professors and university colleagues), as well as to the condition of social isolation and the fear of contagion [14,15,16,17,18,19]. Accordingly, several studies conducted among university students also highlighted that the COVID-19 pandemic and the consequent online learning deeply impacted mental wellbeing [20] and psychological health [21] resulting in poor academic performance [22], academic procrastination [23], and delaying graduation [24].

Therefore, research has underlined a wide range of psychopathological symptoms reported by university students during the pandemic, such as stress, fatigue/exhaustion [25,26,27,28,29], anxiety and phobic anxiety [27,30], obsessive–compulsive disorders [31,32], depression [33,34,35,36], interpersonal sensitivity [37], eating disorders, alcohol and/or substance abuse [38,39,40], and sleep disorders [14,36,41].

In the same direction, longitudinal and repeated cross-sectional studies have been carried out to investigate mental health changes in students across different time points during the pandemic. Most of these studies underlined an increasing trend of students’ psychological disease by considering the time frame closely after the beginning of the pandemic [34,42,43,44]. However, still considering research conducted in 2020, some studies revealed, instead, that psychological symptoms, such as depression [45], anxiety, and obsessive–compulsive disorders [31], decreased after the lifting of the first lockdown. Nevertheless, in 2020, it was not yet expected that the pandemic and the related restrictions would endure further, resulting instead in a long-lasting period of great uncertainty featured by the necessity to paradoxically adjust to a new—unforeseeable—life routine [46], deeply impacting individual and social life domains and potentially triggering or worsening psychological suffering [47,48].

Indeed, in 2021, there was a slight re-opening, followed by a long-lasting succession of full/partial lockdowns (with the constant threat of further/new restrictions). All public places (e.g., universities and libraries) and meeting places (e.g., cinemas, theatres, and restaurants), after being closed for a long time, were gradually re-opened, but they were accessible only with restrictions (e.g., wearing masks, green pass, and curfews). In addition, people were stormed by alarming and sometimes inconsistent information from the mass media (e.g., the number of cases and deaths, the spread of new virus variants, and several issues with the vaccination campaigns). From this perspective, health and ethical implications specifically related to the vaccination campaigns should also be considered. Indeed, the lack of mandatory vaccination and the contradictory mass-media campaign have contributed to individual, relational, and social suffering and resulted in widespread feelings of uncertainty/doubt, hesitancy, anxiety, anger, fear, and social exclusion [49,50,51], with a consequent decrease in vaccination coverage, contributing to the lasting of the COVID-19 emergency. In the same direction, at the individual and relational levels, people had to deal with the abrupt lack of physical contact and the increasing perception of significant others as potential threats (e.g., fear of being infected and infecting relatives) or even “enemies” (vax vs. no-vax). Many persons had to also face their own/significant others’ hospitalizations, as well as losses and grieving [52,53].

Within this context, a repeated cross-sectional study conducted in 2021 [54] explored how university students’ perceived levels of specific COVID-19-related stressors (i.e., relationships and academic life, isolation, and fear of contagion), assessed by using the COVID-19 Student Stress Questionnaire (CSSQ) [18] and psychological health conditions, assessed by using the Symptom Checklist 90 Revised [55,56] evolved over the first year of the pandemic. This study revealed a significant increase in perceived levels of stress related to the changes that occurred in relationships and academic life, the feeling of isolation, and the fear of infection, also underlining a significant exacerbation of psychological symptoms as the pandemic progressed. Furthermore, COVID-19-related stressors emerged as significant predictors of students’ psychopathological risk. These data, thus, reflected the detrimental effects of the first year of the pandemic. 

Afterward, in 2022, the medical emergency ended worldwide, and the control measures related to the pandemic were all lifted. However, nowadays, there is a lack of evidence on the effects of enduring changes experienced over two years of the pandemic, along with a lack of knowledge on the consequences of the end of the critical pandemic time (e.g., returning to the pre-pandemic life routine, potentially entailing not only desires but also concerns). From this perspective, research has well-established the detrimental effects of prolonged feelings of uncertainty and stressful experiences not only in the short-term but also in the long-term [57,58], and research on less-lasting medical emergencies (e.g., H1N1 and SARS) has already warned that the psychological impact of experiences of quarantine and isolation might continue over a long period [59]. Along with this, in the current time, there is an increased concern about psychological disease escalation worldwide [60,61,62], and the “wish” that the effects of the COVID-19 pandemic would have been short-lived and transient seems rather unlikely [63]. Nonetheless, research exploring and comparing perceived stress and psychological health conditions from the beginning of the pandemic to the aftermath of this unique experience is lacking, yet it is needed to provide evidence informing current research and interventions.

Therefore, the present study aims to respond to this need by assessing perceived levels of COVID-19-related stressors (relationships and academic life; isolation; and fear of contagion) and psychophysical symptoms reported by university students in April 2022 (close to the end of the pandemic), comparing data with those registered, respectively, in April 2020 (at the beginning of the pandemic) and in April 2021 (during the pandemic). The role of COVID-19-related stressors in predicting the risk of reporting psychophysical symptoms within the three different times (April 2020; April 2021; and April 2022) was also investigated, providing a more complex overview of the impact of COVID-19-related experiences throughout the pandemic. In line with the study aims, the following research questions were developed and tested:

*Research Question One (RQ1)* is: are there differences in perceived levels of COVID-19-related stressors and psychophysical health conditions reported by university students according to the three study times (April 2020; April 2021; and April 2022)? 

*Research Question Two (RQ2)* is: are there significant associations between perceived COVID-19-related stressors and psychophysical symptoms among university students within the three study times (April 2020; April 2021; and April 2022)? 

## 2. Materials and Methods

### 2.1. Participants and Procedure 

Repeated cross-sectional data were collected among Italian university students over the period from April 2020 to April 2022. This study is part of a larger research that aimed to assess and monitor psychological health conditions among university students from the beginning of the pandemic. Students were asked to participate in an online survey via both institutional channels (e.g., academic mailing lists) and informal channels (e.g., social media groups), and they were given all the information about the research project. Students were provided with all the information about the privacy policy (e.g., the treatment and the confidentiality of their data). Overall, 637 university students (Time 1, April 2020 *n* = 197; Time 2, April 2021 *n* = 200; and Time 3, April 2022 *n* = 240) provided informed consent and completed the survey. The three samples matched for gender and age, with the majority being women (April 2020: Women *n* = 149, 75.6%; April 2021: Women *n* = 151, 75.5%; and April 2022: Women *n* = 181, 75.4%), and the following age means were April 2020: *M* = 21.07, *SD* = 2.74; April 2021: *M* = 20.80, *SD* = 2.88; and April 2022: *M* = 20.93, *SD* = 3.01. There were no missing data.

### 2.2. Ethical Approval 

The study was performed in accordance with the 1964 Helsinki Declaration and its later amendments or comparable ethical standards, and it was approved by an Institutional Review Board (protocol code: 12/2020; date of approval: 12 April 2020).

### 2.3. Measures 

#### 2.3.1. COVID-19-Related Stressors

For the assessment of COVID-19-related Stressors, the COVID-19 Student Stress Questionnaire (CSSQ) [18] was used. The CSSQ consists of 7 items on a 5-point Likert scale ranging from zero “Not at all stressful” to four “Extremely stressful” divided into three subscales: 1. relationships and academic life (4 items, e.g., “How do you perceive the relationships with your university colleagues during this period of the COVID-19 pandemic?!” Cut-off score = 7.69); 2. isolation (2 items, e.g., “How do you perceive the condition of social isolation imposed during this period of the COVID-19 pandemic?” Cut-off score = 5.56); 3. fear of contagion (1 item, i.e., “How do you perceive the risk of contagion during this period of the COVID-19 pandemic?” Cut-off score = 2.73). The mean score value is obtained by summing all the items belonging to each subscale. The scale also provides a global stress score (a sum of all the seven items of the questionnaire, range = 0–28; cut-off score = 14.59; Cronbach’s α = 0.71). The higher the scores, the more the presence of perceived COVID-19-related stressors. Cut-off scores/reliabilities were provided by the Italian [18] validation study.

#### 2.3.2. Psychophysical Symptoms

For the assessment of psychophysical symptoms, the Symptom Checklist 90 Revised (SCL-90-R) [55] (Italian version [56]) was used. The SCL-90-R consists of 90 items on a 5-point Likert scale ranging from zero “Not at all” to four “Extremely” divided into nine subscales: 1. somatization (12 items; Cronbach’s α = 0.83; clinical cut-off men = 1.09, women = 1.67); 2. obsessive–compulsive (10 items; Cronbach’s α = 0.82; clinical cut-off men = 1.41, women = 1.61); 3. interpersonal sensitivity (9 items; Cronbach’s α = 0.83; clinical cut-off men = 1.01, women = 1.34); 4. depression (13 items; Cronbach’s α = 0.87; clinical cut-off men = 1.08, women = 1.62); 5. anxiety (10 items; Cronbach’s α = 0.84; clinical cut-off men = 0.91,women = 1.31); 6. phobic anxiety (7 items; Cronbach’s α = 0.68; clinical cut-off men = 0.44, women = 0.72); 7. hostility (6 items; Cronbach’s α = 0.80; clinical cut-off men = 1.18, women = 1.34); 8. paranoid ideation (6 items; Cronbach’s α = 0.76; clinical cut-off men = 1.00, women = 1.67); 9. psychoticism (10 items; Cronbach’s α = 0.77; clinical cut-off men = 0.71, women = 0.81). The scale also comprises three additional items assessing sleep disorders (3 items; clinical cut-off for both genders = 1.00). For each of the abovementioned subscales, the mean score is calculated by summing all the items belonging to the subscale divided by the number of values (items comprising each subscale). The higher the scores, the more the presence of perceived psychophysical symptoms. Cut-off scores/reliabilities were provided by the Italian [56] validation study. 

### 2.4. Data Analysis

First, to assess and compare perceived levels of COVID-19-related stressors and psychophysical health conditions reported by university students, respectively, in April 2020, April 2021, and April 2022 (Research Question One; RQ1), analysis of variance (ANOVA) tests were used along with Bonferroni’s post hoc tests. Additionally, the study variables were dichotomized into low/high levels referring to the clinical cut-off scores provided by the Italian validation studies (i.e., the CSSQ [18] and the SCL-90-R [56]). Therefore, frequencies/percentages of university students reporting high (and low) levels of COVID-19-related stressors and psychophysical symptoms were calculated, and a comparison across the three study stages was drawn by using cross-tabulations and chi-square (χ^2^) analyses (Research Question One; RQ1). Moreover, in order to explore the associations between COVID-19-related stressors and psychophysical symptoms (Research Question Two; RQ2), respectively, in April 2020, April 2021, and April 2022, logistic regression analyses were carried out (method: enter; entry criterion: *p* < 0.05; removal criterion: *p* > 0.01; first indicator contrast; Hosmer and Lemeshow goodness-of-fit statistic fixed at *p* > 0.05). All the analyses were conducted using the Statistical Package for Social Science (SPSS; Version 21).

## 3. Results

### 3.1. Research Question One (RQ1)

Responding to Research Question One (RQ1; potential differences in perceived levels of COVID-19-related stressors and psychophysical symptoms reported by university students across the three study times, i.e., April 2020; April 2021; and April 2022), Table 1 showed findings from ANOVA and Bonferroni’s post hoc tests (i.e., comparisons of mean and standard deviations scores; Table 1). 

Considering COVID-19-related stressors, the data revealed a statistically significant spike in scores from Time 1—namely from the beginning of the pandemic (April 2020)—to Time 2 (April 2021). However, at Time 3 (April 2022), the registered scores significantly dropped and were also lower than those reported at the beginning of the pandemic (April 2020). Yet, the abovementioned trend concerns the scores registered for perceived stress related to isolation (April 2020 *M* = 3.71; April 2021 *M* = 4.81; and April 2022 *M* = 2.61), fear of contagion (April 2020 *M* = 1.79; April 2021 *M* = 2.59; and April 2022 *M* = 1.23), and global stress (April 2020 *M* = 10.49; April 2021 *M* = 14.01; and April 2022 *M* = 8.62). Differently, albeit decreasing, perceived levels of stress related to changes in relationships and academic life registered in April 2022 were still as high as those reported in April 2020 (April 2020 *M* = 4.99; April 2021 *M* = 6.61; and April 2022 *M* = 4.80).

Considering psychophysical symptoms, the data revealed the same statistically significant spike in scores from Time 1 (April 2020) to Time 2 (April 2021). However, at Time 3 (April 2022), the scores remained fairly steady, specifically for somatization (April 2020 *M* = 0.91; April 2021 *M* = 1.26; and April 2022 *M* = 1.29), interpersonal sensitivity (April 2020 *M* = 0.96; April 2021 *M* = 1.31; and April 2022 *M* = 1.40), depression (April 2020 *M* = 1.24; April 2021 *M* = 1.71; and April 2022 *M* = 1.60), anxiety (April 2020 *M* = 1.03; April 2021 *M* = 1.44; and April 2022 *M* = 1.39), and paranoid ideation (April 2020 *M* = 0.99; April 2021 *M* = 1.33; and April 2022 *M* = 1.35). Differently, the data revealed an ongoing increasing trend for psychoticism symptoms (April 2020 *M* = 0.67; April 2021 *M* = 0.88; and April 2022 *M* = 1.02). No other statistically significant differences across the three study times were found (Table 1). 

Still responding to research question one (RQ1), Table 2 showed findings from cross-tabulations and chi-square analyses (comparisons of frequencies and percentages of university students reporting low and high/clinically relevant levels of perceived COVID-19-related stressors and psychophysical symptoms across the three study times; Table 2). 

Considering COVID-19 related stressors, the data substantially confirmed findings from ANOVA, revealing a remarkable increase—from April 2020 to April 2021—and a sharp decrease—in April 2022—in the number of students reporting clinically relevant levels of fear of contagion (April 2020 *n* = 61, 30.9%; April 2021 *n* = 118, 59%; and April 2022 *n* = 31, 13.0%), isolation (April 2020 *n* = 47, 23.9%; April 2021 *n* = 78, 39%; and April 2022 *n* = 26, 10.7%), and global stress (April 2020 *n* = 39, 19.8%; April 2021 *n* = 90, 45%; and April 2022 *n* = 36, 15.1%). Differently, albeit decreasing from April 2021, the number of students reporting high levels of relationships and academic life in April 2022 was higher than those registered at the beginning of the pandemic (April 2020 *n* = 31, 15.7%; April 2021 *n* = 76, 38%; and April 2022 *n* = 53, 22%). 

Considering psychological health conditions, the data provided more strike evidence of the increase in psychophysical symptoms, with a sharp, ongoing, and statistically significant growth—from April 2020 to April 2022—in the number of students reporting clinically relevant levels of somatization (April 2020 *n* = 33, 17%; April 2021 *n* = 65, 32.5%; and April 2022 *n* = 96, 39.7%), interpersonal sensitivity (April 2020 *n* = 50, 25.4%; April 2021 *n* = 93, 46.5%; and April 2022 *n* = 121, 50.2%), anxiety (April 2020 *n* = 67, 34%; April 2021 *n* = 93, 46.5%; and April 2022 *n* = 119, 49.8%), and paranoid ideation (April 2020 *n* = 48, 24.4%; April 2021 *n* = 74, 37%; and April 2022 *n* = 105, 43.8%). Differently, the number of students reporting clinically relevant levels of depression (April 2020 *n* = 75, 38.1%; April 2021 *n* = 115, 57.5%; and April 2022 *n* = 134, 55.6%) and psychoticism (April 2020 *n* = 69, 35%; April 2021 *n* = 110, 55%; and April 2022 *n* = 128, 53.4%) notably raised from April 2020 to April 2021, yet it slightly decreased in April 2022.

Furthermore, despite the non-significance of comparisons across the three study times, alarming frequencies/percentages of students reporting clinically relevant levels of sleep disorders (April 2020 *n* = 134, 68.1%; April 2021 *n* = 134, 67%; and April 2022 *n* = 136, 56.9%), obsessive–compulsive symptoms (April 2020 *n* = 75, 38.1%; April 2021 *n* = 100, 50%; and April 2022 *n* = 115, 47.8%), phobic anxiety (April 2020 *n* = 50, 25.4%; April 2021 *n* = 83, 41.5%; and April 2022 *n* = 82, 34.3%), and hostility (April 2020 *n* = 46, 23.4%; April 2021 *n* = 54, 27%; and April 2022 *n* = 79, 32.7%) were also registered (Table 2).

### 3.2. Research Question Two (RQ2)

Responding to Research Question Two (RQ2; significant associations between perceived COVID-19-related stressors and psychophysical symptoms among university students within the three study times, i.e., April 2020; April 2021; and April 2022), Table 3 displayed findings from logistic regression analyses. 

Specifically, the data revealed that students who perceived high levels of stress related to relationship and academic life were at significantly higher risk for reporting clinically relevant levels of the following psychophysical symptoms: anxiety in April 2020; all psychophysical symptoms except for paranoid ideation in April 2021; and somatization, phobic anxiety, and sleep disorders in April 2022. 

Moreover, students who perceived high levels of stress related to isolation were at significantly higher risk for reporting clinically relevant levels of the following psychophysical symptoms: somatization and hostility in April 2020; somatization, obsessive–compulsive, anxiety, depression, and psychoticism in April 2021; and anxiety and sleep disorders in April 2022.

Finally, students who perceived high levels of stress related to fear of contagion were at significantly higher risk for reporting clinically relevant levels of the following psychophysical symptoms: interpersonal sensitivity, anxiety, and phobic anxiety in April 2020; obsessive–compulsive and phobic anxiety in April 2021; and obsessive–compulsive, anxiety, depression, sleep disorders, and psychoticism in April 2022 (Table 3).

## 4. Discussion

The present study aimed to provide a comprehensive overview of the psychological impact of the last several years of unprecedented challenges imposed by the pandemic emergency on university students’ lives, targeting three specific and different time frames, i.e., April 2020, namely at the beginning of the outbreak and containment measures; April 2021, namely after one year of long-lasting and drastic changes and challenges in both customary and academic life; and April 2022, namely, at the end of the emergency. 

Nowadays, indeed, COVID-19 has no longer been defined as a Public Health Emergency of International Concern (PHEIC) for a while, yet there is still a need to advance knowledge and understanding of people’s experiences throughout different phases of this overwhelming event as well as in the aftermath. The present study sought to respond to this—still high—need by targeting the specific experiences of university students.

Despite the cross-sectional nature of our study design and the gender imbalance of our study sample, the overall findings provided evidence-based insights into the detrimental effects of COVID-19-related experiences. Specifically, by responding to the first research question (RQ1), the study provided tailored evidence on the specificity of significant changes (peaks and declines) in stress levels and psychophysical health conditions over the two years of the pandemic and at its conclusion. Specifically, the findings were united to confirm previous evidence underlining the detrimental effects of the first year of the pandemic [35,43,44,54], with a significant spiking of both COVID-19-related stressors and psychophysical symptoms from the beginning (2020) to the conclusion of the first year of the pandemic (2021).

Otherwise, the data registered in 2022—namely at the end of the pandemic emergency—underlined two distinguished paths, one for COVID-19-related stressors and the other one for psychophysical health. Specifically, concerning COVID-19-related stressors, the end of the pandemic entailed a substantial and clear drop in stress related to isolation and fear of contagion, while—interestingly—stress related to relationships and academic life was still at a noteworthy high in April 2022. 

Overall, these data seem to capture the positive effects of the lifting of all the restrictions (i.e., ending of prolonged and mandatory isolation due to lockdowns, curfews, and social distancing) and of the medical advancements (i.e., lowering of the fears to be infected/infecting others due to the higher control over the treatment, the vaccination campaigns, and the availability of COVID-19-specific drugs). From this perspective, the changes that emerged in perceived stressors in 2022 and, specifically, the reduction of perceived stress related to isolation and fear of contagion, could also be partially explained by the re-opening (quite permanently for a while) of all places and the restoration of face-to-face contacts and activities.

However, the lack of changes (reduction) in stress related to relationships and academic life requires a tailored reflection. Indeed, our data highlighted the potential marks and scars of the long-lasting challenges of in-person relational experiences at the end of the emergency, namely in April 2022. Yet, these data emphasize the key role that the relational life (i.e., relationships with members of one’s family of origin, with other adults, such as academic professors, and with peers) play in people’s development and flourishing, mainly among the delicate phase of emerging adulthood [64]. 

From this perspective, the abrupt advent of the COVID-19 pandemic has undoubtedly resulted in a significant disruption in students’ developmental path, entailing relational, social, and academic life. Primarily, the intensification of relationships with family members (i.e., sharing all the spaces and time when cohabitants), and the simultaneously prolonged detachment from peers and the university community may have determined long-lasting difficulties and flaws in the relational area. Additionally, the challenges of online learning (i.e., technostress) [35,65] and the potential frustration linked to the lack of possibility of enjoying—“as everyone else”—the academic community life may have also contributed to strengthening the perceived stress linked to this specific dimension. These data could, however, also reflect the efforts spent by students to re-adjust to “face-to-face” social life and (re-)join the university community. This might be especially true for those students who started university life with distance learning; thus, they may have never or only partially benefited from academic life, both formal (e.g., exams and appointments with professors) and informal (e.g., use of study halls and coffee breaks with colleagues). 

However, whereas perceived levels of COVID-19-related stressors substantially decreased as a result of the end of the emergency, the findings revealed a sharp and ongoing increase in psychophysical suffering, mainly in terms of somatization and interpersonal sensitivity, and also of paranoid ideation and psychoticism. These findings are in line with previous research underlining the significant psychological impact of the COVID-19 emergency among university students [25,26,27,28,29,30,31,32,33,34,35,36,37,38,39,40,41,66], and they provide evidence sustaining the studies conducted at the beginning of the pandemic, which already warned about the increasing psychological risk among young adults/students as the pandemic was progressing [42,43,44]. 

Additionally, our data also emphasized an interesting change in the painting of the symptoms. Specifically, at the beginning of the pandemic (2020), our data revealed the predominant presence of clinically relevant levels of sleep disorders (68.1% of students), probably also connected to the sudden change in their daily routine/schedule and, most of all, due to the greater use of technological devices that are well-recognized as being able to reduce the sleep quality (i.e., increasing the sense of excitement/tension; prolonging the sleep-onset latency; and reducing sleep duration) [67]. Yet, in 2021, the unexpected persistence of the pandemic emergency seems to have led to a substantial and rather pervasive increase in psychological suffering, resulting in the wide spread of several different symptoms. In 2022, however, once the emergency was over, specific symptoms seem to have stood out (continued to grow), namely anxiety and somatization, along with paranoid ideation, interpersonal sensitivity, and hostility, which could align with the challenging experiences of uncertainty and relational difficulties and the adjustment efforts to get ” back to reality”.

Notwithstanding, overall, the data registered in 2022 revealed remarkable clinically relevant levels of anxiety, phobic anxiety, and obsessive–compulsive disorders, probably linked to the overload of long-lasting recommendations for strict hygiene measures and ritualistic patterns (e.g., repeated washing) and the normalization of behaviors such as avoiding crowded places and ruminating thoughts of contamination [68]. 

Furthermore, astoundingly, our data revealed that, at the end of the pandemic (April 2022), more than one-half of university students reported clinically relevant levels of depression and sleep disorders, interpersonal sensitivity, and psychoticism and more than 40% reported clinical levels of paranoid ideation. The above-mentioned psycho-pathological portrait could be (at least partially) explained by the extreme experiences of withdrawal/isolation and prolonged loss of contact (mainly physical) with others, as well as the drastic changes in students’ daily routines who needed to adjust to the new—unrecognizable—reality (e.g., beliefs that COVID-19 pandemic cannot be real).

From this perspective, it should be noted that the percentages of students reporting clinical levels of psychoticism (53.4%), interpersonal sensitivity (50.2%), and paranoid ideation (43.8%) registered in April 2022 were too high to be explained only by the idea that the onset of psychotic symptoms and schizophrenia matches with the start of university life [1,10,11], requiring the framing of them in the context of the COVID-19-related experiences. In line with this, our data seem to align with the recent evidence on the significant bi-directional relationship between loneliness and psychotic experiences among university students, mainly with the idea that feeling of isolation may lead to maladaptive cognition of both oneself and others [69]. 

Overall, considering the abovementioned findings (RQ1), there is a clear implication for current research and interventions targeting the psychological health of young adults—and in particular of university students—namely, there is an urgent need to carefully take into account not only the alarming psychopathological risk highlighted among young adults/students before the pandemic [1,2,3,4,5,6,7], but to also consider the warning portrait and the observed increasing trend of relational difficulties and psychophysical suffering gathered at the end of the pandemic.

From this perspective, by responding to the second research question (RQ2), the study provides further evidence endorsing this urgent need. A comprehensive overview of the specific paths of associations between COVID-19-related stressors and psychophysical symptoms was provided, allowing for the estimation of the psychopathological risks students have been exposed to during the last several years. In particular, referring to the end of the emergency (2022), the data revealed that students who still perceived high stress related to relationships and academic life and isolation, despite the re-opening of universities and the re-establishing of the pre-pandemic life routines, were at significant risk, specifically in terms of somatization, anxiety, and sleep disorders. These data should be carefully considered in light of the pre-existing risk of isolation/loneliness reported in young people, especially college students [7], requiring even further efforts to effectively support their interpersonal skills at the current time. 

Moreover, the data revealed that students who—in April 2022—still perceived high stress related to fear of contagion, despite the vaccination campaign and the advances in medicine, reported a greater psychopathological risk, as they were at significantly higher risk for reporting anxiety and obsessive–compulsive (e.g., repeated/compulsive hand-washing and other compulsive behaviors and obsessive thoughts), depression, and sleep disorders (e.g., withdrawal, defensive avoiding behaviors, and insomnia/hypersomnia), as well as psychoticism (e.g., distorted reality and loss of reality, disturbances of thinking and feelings). These findings can be useful for understanding, through evidence-based information, the potentially deleterious impact of specific stressors related to the pandemic even after it ends (April 2022), which seems able to even trigger persecution fantasy. Otherwise, the mass-media coverage and the difficulties related to the vaccination campaign (e.g., conflicting information, contraindications, and deaths) [50] could also reinforce the uncertainty, thus probably explaining the presence of the psychopathological risk linked to the fear of contagion even after two years from the beginning of the pandemic. 

Based on this evidence, researchers, stakeholders in higher education (HE), and clinicians may be warned about the need to carefully consider that the traumatic experiences and prolonged extreme challenges—such as those imposed by the pandemic—can have detrimental effects, whether they are clear or hidden, both in the short and in the long term [70,71]. Indeed, in the current period, specific psychophysical symptoms might be more frequently reported by young adults/university students, such as clinically relevant levels of anxiety, relational concerns, remarkable and persistent sleep disturbances, obsessive–compulsive symptoms, or even widespread feelings of detachment from reality. These symptoms should be assessed and framed by also considering the two pandemic years (from 2020 to 2022) since the pandemic experience has entailed—undoubtedly—a severe disruption in a delicate phase of their developmental life. In line with this, recent studies also highlighted a wide variety of outcomes expressing students’ suffering in the academic context, such as apathy, disengagement, decreased focus and motivation [65,72,73,74,75,76,77], delaying graduation, increasing academic procrastination, and worries about their career trajectory [20,24]. 

From this perspective, although we are nowadays living in completely changed circumstances, higher education (HE) administrators could schedule screening and monitoring of students’ mental health, as well as foster exchanges with them to achieve a greater understanding of their current needs. 

Furthermore, mental health professionals could also benefit from our findings, which suggest the need to pay further attention to the exploration of the relational changes that occurred during these years, yet are inscribed within a specific moment in life, namely emerging adulthood, which represents an already complex period due to the changes in family dynamics, the further explorations of relationships with peers, and the building of new relationships related to academic life. 

Nonetheless, interventions should not only target students who reported they have perceived remarkable levels of COVID-19-related stressors during the emergency but also those who may perceive lowered stress. Indeed, the significant psychopathological portrait registered in April 2022 suggests that students may report notable psychophysical disorders due to past COVID-19-related experiences, which have indeed required them to spend much effort to adjust and recover from this unique long-lasting condition. In particular, the data revealed a significant increasing trend for psychophysical disorders (i.e., somatization), as well as relational disorders (i.e., interpersonal sensitivity, paranoid ideation, and psychoticism). Future research should, therefore, keep assessing students’ psychological health, starting with the need to target these specific diseases.

Moreover, as further implications for practitioners, the psychopathological profiles that emerged from our data suggest the need for mental health professionals to routinely explore COVID-19-related past experiences, even when an explicit link with the current discomfort seems lacking. The hidden traces of these years of drastic changes could be key elements to understanding patients’ diseases (e.g., phobic anxiety, obsessive–compulsive disorders, and psychoticism) and, accordingly, to define more effective interventions.

However, despite the fact that our study may potentially have several implications for research and interventions, some limitations need to be acknowledged. First, data were repeated cross-sectional, so limiting the possibility of making causal relations, and no inferences about the temporal associations between predictors and outcomes can be suggested. Future research could be developed with a longitudinal design, with a long-term follow-up design, to assess and monitor over time the psychological health conditions of vulnerable populations, such as young adults/university students, and to further identify potential long-lasting effects of the pandemic experience. Second, data were self-reported so they may have been more vulnerable to social desirability biases. Third, the size of our sample is not excessively large, and the participant pool comprised a convenience sample of students with a majority being female, thus limiting the generalizability of the results; further investigation on bigger and more representative samples is, therefore, needed to confirm the findings highlighted in the present study. Finally, the study variables selected were COVID-19-related stressors and psychophysical symptoms. Yet, other factors, such as resources (e.g., social support and coping strategies) or some potential confounding (e.g., experiences with a mental health service/professional and participation in programs to support students’ mental health) have not been considered. Future research will therefore aim at exploring a wider set of study variables potentially influencing students’ psychophysical health. From this perspective, future research should also consider the need to address the potential role of external factors, such as the impact of online learning and technology or mandatory social isolation measures. This could offer more targeted recommendations for academic institutions. In line with this, future research could also explore the impact of specific interventions to support students’ mental health in the post-pandemic recovery phase. This would give further and tailored indications to be adopted in developing evidence-based programs and interventions.

## 5. Conclusions

Despite the abovementioned limitations, the findings provided evidence-based and tailored insights into the detrimental effects of COVID-19-related experiences on students’ psychophysical health conditions. In summary, the findings suggested that the end of the pandemic was featured by a substantial drop in perceived stress related to isolation and fear of contagion, while the deep challenges experienced in relational and academic life required higher adjustment efforts by students. The findings also indicated an ongoing increase in psychophysical suffering reported by students, mainly in terms of somatization and interpersonal sensitivity, but also of paranoid ideation and psychoticism. Moreover, referring to data collected at the end of the emergency (2022), the data revealed that students who still perceived noteworthy levels of stress related to the COVID-19 experience were at significant psychopathological risks.

The findings can inform the development of future research on stress and health processes in the post-pandemic era and can be also useful in implementing evidence-based interventions to effectively support students in dealing with the complex post-emergency adjustment process. Indeed, the end of the global emergency should not be considered as an occasion “to pack up and move on” [78], rather it calls to action addressing the marks and the lessons learned from the past years.

## Figures and Tables

**Table 1:** COVID-19-related stressors and psychophysical symptoms reported by university students: comparisons of means (M) and standard deviations (SD) across the three study times (April 2020, April 2021, April 2022).

	April-2020 *n* = 197	April-2021 *n* = 200	April-2022 *n* = 240	ANOVA *F*	Comparison (s) ^a^
	*M* ± *SD*	*M* ± *SD*	*M* ± *SD*		
**Perceived COVID-19-related stressors**					
Relationships and Academic Life (CSSQ-REL)	4.99 ± 2.58	6.61 ± 3.21	4.80 ± 3.31	12.13 ***	T2 > T1 **, T3 ***
Isolation (CSSQ-ISO)	3.71 ± 2.00	4.81 ± 2.03	2.61 ± 2.07	42.33 ***	T2 > T1 **, T3 ***; T3 < T1 ***
Fear of Contagion (CSSQ-FEAR)	1.79 ± 1.22	2.59 ± 1.13	1.23 ± 1.09	51.61 ***	T2 > T1 ***, T3 ***; T3 < T1 ***
Global Stress (CSSQ-GLOBAL)	10.49 ± 4.32	14.01 ± 4.85	8.62 ± 5.38	39.98 ***	T2 > T1 ***, T3 ***; T3 < T1 **
**Psychophysical Symptoms**					
Somatization (SOM)	0.91 ± 0.69	1.26 ± 0.84	1.29 ± 0.97	6.50 **	T1 < T2 *, T3 **
Obsessive–Compulsive (O-C)	1.46 ± 0.74	1.59 ± 0.81	1.59 ± 0.86	0.88	-
Interpersonal Sensitivity (INT)	0.96 ± 0.65	1.31 ± 0.71	1.40 ± 0.86	10.18 ***	T1 < T2 **, T3 ***
Depression (DEP)	1.24 ± 0.71	1.71 ± 0.83	1.60 ± 0.91	8.39 ***	T1 < T2 **, T3 **
Anxiety (ANX)	1.03 ± 0.70	1.44 ± 0.82	1.39 ± 0.96	6.42 **	T1 < T2 **, T3 **
Hostility (HOS)	0.92 ± 0.67	1.11 ± 0.76	1.14 ± 0.92	2.44	-
Phobic Anxiety (PHOB)	0.51 ± 0.58	0.72 ± 0.66	0.69 ± 0.77	2.63	-
Paranoid Ideation (PAR)	0.99 ± 0.69	1.33 ± 0.77	1.35 ± 0.90	6.62 **	T1 < T2 *, T3 **
Psychoticism (PSY)	0.67 ± 0.44	0.88 ± 0.62	1.02 ± 0.80	7.84 ***	T1 < T3 ***
Sleep Disorders (SLEEP)	1.35 ± 0.99	1.42 ± 1.06	1.36 ± 1.15	0.11	-

^a^ Bonferroni post hoc test. T1, April 2020; T2, April 2021; T3, April 2022. * *p* < 0.05. ** *p* < 0.01. *** *p* < 0.001.

**Table 2:** Low and high/clinically relevant levels of COVID-19-related stressors and psychophysical symptoms reported by university students: Comparisons of frequencies (N) and percentages (%) across the three study times (April 2020, April 2021, April 2022).

	April-2020 *n* = 197	April-2021 *n* = 200	April-2022 *n* = 240	Chi-Square
	*N* (%)	*N* (%)	*N* (%)	χ^2 a^
**Perceived COVID-19-related stressors**				
Relationship and Academic Life (CSSQ-REL)				
Low	166 (84.3)	124 (62.0)	187 (77.9)	
High	31 (15.7)	76 (38.0)	53 (22.1)	14.92 **
Isolation (CSSQ-ISO)				
Low	150 (76.1)	122 (61.0)	214 (89.3)	
High	47 (23.9)	78 (39.0)	26 (10.7)	35.83 ***
Fear of Contagion (CSSQ-FEAR)				
Low	136 (69.1)	82 (41.0)	209 (87.0)	
High	61 (30.9)	118 (59.0)	31 (13.0)	75.20 ***
Global Stress (CSSQ-GLOBAL)				
Low	158 (80.2)	110 (55.0)	204 (84.9)	
High	39 (19.8)	90 (45.0)	36 (15.1)	36.08 ***
**Psychophysical Symptoms**				
Somatization (SOM)				
Low	164 (83.0)	135 (67.5)	144 (60.3)	
High	33 (17.0)	65 (32.5)	96 (39.7)	15.08 **
Obsessive–Compulsive (O-C)				
Low	122 (61.9)	100 (50.0)	125 (52.2)	
High	75 (38.1)	100 (50.0)	115 (47.8)	3.09
Interpersonal Sensitivity (INT)				
Low	147 (74.6)	107 (53.5)	119 (49.8)	
High	50 (25.4)	93 (46.5)	121 (50.2)	16.50 ***
Depression (DEP)				
Low	122 (61.9)	85 (42.5)	106 (44.4)	
High	75 (38.1)	115 (57.5)	134 (55.6)	9.23 *
Anxiety (ANX)				
Low	130 (66.0)	107 (53.5)	121 (50.2)	
High	67 (34.0)	93 (46.5)	119 (49.8)	6.51 *
Hostility (HOS)				
Low	151 (76.6)	146 (73.0)	161 (67.3)	
High	46 (23.4)	54 (27.0)	79 (32.7)	2.94
Phobic Anxiety (PHOB)				
Low	147 (74.6)	117 (58.5)	158 (65.7)	
High	50 (25.4)	83 (41.5)	82 (34.3)	5.21
Paranoid Ideation (PAR)				
Low	149 (75.6)	126 (63.0)	135 (56.2)	
High	48 (24.4)	74 (37.0)	105 (43.8)	10.37 **
Psychoticism (PSY)				
Low	128 (65.0)	90 (45.0)	112 (46.6)	
High	69 (35.0)	110 (55.0)	128 (53.4)	9.95 **
Sleep Disorders (SLEEP)				
Low	63 (31.9)	66 (33.0)	104 (43.1)	
High	134 (68.1)	134 (67.0)	136 (56.9)	4.81

^a^ Cross-tabulations and chi-square analyses. * *p* < 0.05. ** *p* < 0.01. *** *p* < 0.001.

**Table 3:** Associations between COVID-19-related stressors and risk for reporting clinically relevant levels of psychophysical symptoms among university students across the three study times (April 2020; April 2021; and April 2022).

		April-2020			April-2021			April-2022	
	CSSQ-REL	CSSQ-ISO	CSSQ-FEAR	CSSQ-REL	CSSQ-ISO	CSSQ-FEAR	CSSQ-REL	CSSQ-ISO	CSSQ-FEAR
	*OR* (*C.I.*)	*OR* (*C.I.*)	*OR* (*C.I.*)	*OR* (*C.I.*)	*OR* (*C.I.*)	*OR* (*C.I.*)	*OR* (*C.I.*)	*OR* (*C.I.*)	*OR* (*C.I.*)
**SOM**	2.0 (0.5–7.4)	6.4 (2.0–20.4) **	1.4 (0.5–4.4)	4.7 (1.8–11.9) **	2.7 (1.1–6.7) *	2.2 (0.9–5.5)	1.9 (1.0–3.8) *	1.7 (0.7–4.2)	1.5 (0.6–3.3)
**O-C**	2.8 (0.9–8.9)	1.5 (0.6–3.9)	1.0 (0.4–2.4)	5.7 (2.2–14.7) ***	2.8 (1.2–6.6) *	2.7 (1.2–6.4) *	1.4 (0.7–2.7)	1.6 (0.6–4.1)	2.9 (1.2–7.0) *
**INT**	2.3 (0.7–7.2)	0.8 (0.3–2.5)	3.1 (1.2–8.2) *	2.4 (1.0–5.7) *	1.6 (0.7–3.8)	0.7 (0.3–1.7)	1.2 (0.6–2.3)	2.2 (0.9–5.8)	1.9 (0.8–4.5)
**DEP**	2.9 (0.9–8.9)	1.1 (0.4–3.1)	1.5 (0.6–3.6)	15.7 (4.3–57.3) ***	4.7 (1.8–12.1) **	1.1 (0.4–2.5)	1.6 (0.8–3.2)	2.2 (0.8–6.1)	3.2 (1.2–8.4) *
**ANX**	5.2 (1.6–16.9) **	1.5 (0.5–3.9)	3.0 (1.2–7.5) *	2.4 (1.0–5.8) *	3.5 (1.5–8.4) **	1.5 (0.6–3.4)	1.6 (0.8–3.0)	2.9 (1.1–7.7) *	2.3 (1.0–5.5) *
**HOST**	1.2 (0.3–4.3)	3.1 (1.1–8.9) *	1.1 (0.4–3.0)	3.2 (1.2–8.3) *	1.9 (0.8–4.9)	0.6 (0.2–1.5)	1.4 (0.7–2.8)	2.1 (0.8–5.2)	0.9 (0.4–2.2)
**PHOB**	1.6 (0.5–5.2)	2.0 (0.7–5.6)	5.1 (1.9–13.8) **	3.5 (1.4–8.5) **	1.7 (0.7–3.9)	4.0 (1.6–10.1) **	2.1 (1.1–4.2) *	1.9 (0.8–4.8)	1.1 (0.5–2.5)
**PAR**	0.7 (0.2–2.9)	0.9 (0.3–2.7)	0.9 (0.3–2.7)	2.1 (0.9–5.2)	1.6 (0.6–3.7)	0.6 (0.3–1.4)	0.8 (0.4–1.5)	1.5 (0.6–3.7)	0.9 (0.4–2.1)
**PSY**	1.3 (0.4–4.0)	1.8 (0.7–4.7)	1.8 (0.7–4.6)	9.0 (3.3–24.9) ***	3.2 (1.4–7.6) **	0.9 (0.4–2.1)	1.7 (0.8–3.4)	1.2 (0.5–3.0)	2.4 (1.0–5.8) *
**SLEEP**	1.2 (0.3–4.2)	0.9 (0.3–2.6)	0.8 (0.3–2.1)	4.1 (1.5–11.2) **	1.8 (0.7–4.2)	1.6 (0.7–3.7)	2.9 (1.4–6.0) **	4.4 (1.5–13.4) **	3.6 (1.3–10.1) *

*OR* = odds ratio; *C.I.* = confidence interval; * *p* < 0.05. ** *p* < 0.01. *** *p* < 0.001.

## Data Availability

The data that support the results of this study are available from the corresponding author upon reasonable request.
